# The Activation of PKM2 Induces Pyroptosis in Hippocampal Neurons via the NLRP3/Caspase‐1/GSDMD Pathway in Neonatal Rats With Hypoxic‐Ischemic Brain Injury

**DOI:** 10.1002/brb3.70108

**Published:** 2024-10-23

**Authors:** Sha Sha, Ni Jin, Ruiyu Zhou, Yanghao Ruan, Ying Ouyang

**Affiliations:** ^1^ Sun Yat‐sen Memorial Hospital Sun Yat‐Sen University Guangzhou China; ^2^ The Fifth Affiliated Hospital of Guangzhou Medical University Guangzhou Guangdong China; ^3^ The Fifth Affiliated Hospital of Sun Yat‐sen University Zhuhai Guangdong China

**Keywords:** hypoxic‐ischemic brain damage, NLRP3/Caspase‐1/GSDMD, PKM2, pyroptosis

## Abstract

**Introduction:**

The presence of hypoxic‐ischemic brain damage (HIBD) in neonates triggers a strong neuroinflammatory reaction. Pyroptosis, a programmed cell death mechanism associated with inflammation, plays a crucial role in HIBD. Pyruvate kinase M2 (PKM2) plays a significant role in connecting metabolic processes and inflammatory responses, but whether it affects hippocampus pyroptosis in HIBD is unclear. The aim of this study is to elucidate the role of PKM2 in HIBD and to propose a novel therapeutic approach for neonatal ischemic‐hypoxic encephalopathy.

**Methods:**

In this study, we employed neonatal 7‐day‐old Sprague Dawley rats to establish a model of HIBD using the Rice–Vannucci surgical technique and a hypoxia device. To inhibit the elevation of PKM2, we utilized the PKM2 inhibitor shikonin. The rats were categorized into four groups: Sham, Shikonin, HIBD, and Shikonin + HIBD. Behavioral tests, hematoxylin eosin staining, immunofluorescence staining, ELISA (IL‐1β, IL‐18), and LDH were conducted in each group to evaluate neurological function, hippocampal damage, the occurrence of neuronal pyroptosis, and the neuroinflammation. Western blot was used to assess the expression levels of PKM2, NLRP3, Caspase‐1, Cleaved Caspase‐1, GSDMD, GSDMDN, and IL‐1β.

**Results:**

The expression of PKM2 elevated in hippocampal tissues of the HIBD model and the localization of PKM2 in the hippocampus was activated in neurons instead of microglia during the HIBD. Meanwhile, the inhibition of PKM2 improved the behavioral test scores and the body weight of rats, the neuronal damage in the CA1 region of hippocampal tissue was also attenuated. In addition, inhibiting PKM2 alleviated neuronal pyroptosis by decreasing the expression of PKM2, NLRP3, Caspase‐1, Cleaved Caspase‐1, GSDMD, GSDMDN. Furthermore, serum levels of LDH and inflammatory factors IL‐1β and IL‐18 decrease with PKM2 inhibition.

**Conclusions:**

Based on these findings, we can conclude that PKM2 plays a crucial role in regulating hippocampal neuronal pyroptosis of HIBD rats via NLRP3/Caspase‐1/GSDMD pathway. Therefore, inhibiting PKM2 could be a promising therapeutic strategy for the treatment of neonatal ischemic‐hypoxic encephalopathy.

## Graphical abstract

In this study, we observed an upregulation of PKM2 expression in the hippocampal tissues of the HIBD model. Inhibition of PKM2 resulted in a reduction of neuronal damage in the CA1 region of the hippocampus, alongside improvements in behavioral test outcomes and body weight gain in rats. Moreover, the inhibition of PKM2 mitigated neuronal pyroptosis possibly by downregulating the expression of NLRP3/Caspase‐1/GSDMD pathway (By Figdraw ID:TIAIPb2442)

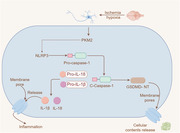

## 1 Introduction

During the perinatal period, neonates are at risk of experiencing hypoxic‐ischemic brain damage (HIBD) due to a significant reduction in cerebral oxygen and/or blood supply (Mattiesen et al. 2009). Research has indicated that newborns who have undergone asphyxia and subsequently developed HIBD have a predicted mortality rate ranging from 20% to 25%. In addition, an additional 25% of these individuals are likely to face long‐term challenges, including conditions such as cerebral palsy, epilepsy, autism spectrum disorders, and cognitive impairments (Dixon et al. 2015). Currently, therapeutic hypothermia is the established and extensively employed intervention for infant HIBD (Douglas‐Escobar and Weiss 2015; Jacobs et al. 2013). Nevertheless, the neuroprotective mechanisms underlying this therapy are intricate and encompass the reduction of cerebral metabolic energy demand, mitigation of inflammation, oxidative and excitotoxic harm, and attenuation of apoptosis (Yenari and Han 2012). Despite its ability to enhance survival and neurological development in newborns with HIBD, the advantages gradually diminish over time due to the limited time frame for application, incompleteness, and variations in individual efficacy (Romeo et al. 2019; Dereymaeker et al. 2019). Hence, scholars are currently investigating alternative neuroprotective medications as adjunctive and alternative measures.

The immune response and neuroinflammation play a significant role in the delayed neuronal death that occurs after a stroke (Maida et al. 2020). In newborns, HIBD triggers a robust neuroinflammatory response, typically leading to a worsening prognosis (Bernis et al. 2023). Pyroptosis, a recently discovered form of programmed cell death, relies on cysteine‐dependent aspartate‐specific proteases (cystatinases) and involves the formation of distinct “inflammatory vesicles.” The GSDMD‐forming spliceosome, GSDMDN, ultimately induces cellular perforation, resulting in cell death and the release of substantial amounts of inflammatory substances (Barnett et al. 2023; Zhang et al. 2020). Recent studies indicate that inhibiting the activation of the pyroptosis pathway could effectively suppress the inflammatory cascade response, leading to a reduction in ischemic brain injury (Gou et al. 2021). Previous studies have suggested that, in the context of spinal cord injury, neuronal apoptosis tends to peak before the initiation of neuronal pyroptosis (Li et al. 2020). Moreover, it has been observed that the duration of pyroptosis surpasses that of apoptosis to a considerable extent. The NLRP3/Caspase‐1/Gasdermin‐D (GSDMD) axis is the classical pyroptosis pathway. A new study found that the NLRP3/Caspase‐1/GSDMD axis‐mediated microglial cell pyroptosis plays a key role in neonatal HIBD (Lv et al. 2020). Consequently, it can be deduced that cellular pyroptosis may also play a crucial role in the pathogenesis of neonatal HIBD. Notably, research on cellular pyroptosis caused by ischemic‐hypoxic encephalopathy in neonatal rats mainly focuses on microglia, and the precise mechanism of neuronal pyroptosis is still unclear.

Gene expression profiles of human brain tissue organs indicate that pyruvate kinase M2 (PKM2) is a key marker of oxygen–glucose deprivation and reoxygenation (OGD/R) (Iwasa et al. 2021). PKM2 is not only a key rate‐limiting enzyme of glycolysis, but also a transcriptional target of several proinflammatory mediators (Romero‐Ramírez et al. 2022). It is mainly found in proliferating cells, especially neural progenitors in embryonic cells, hippocampus, cerebellum, and subventricular region. Among them, the hippocampus has an important influence on cognitive functions such as spatial memory and learning ability of the brain. Immature hippocampal neurons are particularly sensitive to hypoxic and ischemic states, and their damage is closely associated with neurological sequelae after HIE (Bird and Burgess 2008). PKM2 can play a role in a variety of neurological disorders by regulating glycolysis, inflammatory responses, apoptosis, cell proliferation, oxidative stress, mitochondrial dysfunction, or pathological autoimmune responses (Romero‐Ramírez et al. 2022), such as Alzheimer's disease, cognitive dysfunction, ischemic stroke, poststroke depression, cerebral small vessel disease, and hypoxic‐ischemic encephalopathy (Tech et al. 2017). PKM2 plays an important role in connecting metabolic processes and inflammatory responses (Israelsen et al. 2013; Long et al. 2020). Studies have shown that nuclear PKM2 is upregulated in neutrophils and promotes neutrophil hyperactivation after ischemic stroke attacks. Meanwhile, PKM2 deficiency in myeloid cells improves stroke outcomes by limiting post‐ischemic cerebral thrombotic inflammation. Shikonin (PKM2 inhibitor) has a potential therapeutic role in modulating thrombotic inflammation in PKM2‐deficient mice with cerebral ischemia and reperfusion injury (Dhanesha et al. 2022). In addition, PKM2‐mediated aerobic glycolysis stimulates NLRP3 in macrophages, leading to the synthesis of proinflammatory cytokines such as IL‐1β (Zhang et al. 2020). It is suggested that PKM2 may be involved in neuroinflammatory responses by regulating NLRP3. However, whether PKM2 is involved in ischemia‐hypoxia‐induced neuronal focal death in neonatal rats and its related mechanisms remain unknown.

This study aimed to examine the impact of heightened PKM2 levels on neurological function and hippocampus damage in rats with HIBD. By employing ischemic hypoxia modeling on neonatal rats, we identified for the first time the cellular localization of elevated PKM2 in the hippocampal tissue of HIBD rats. In addition, we observed the augmented PKM2, NLRP3/Caspase‐1/GSDMD pathway expression in hippocampal neurons of HIBD rats, the elevated cellular pyroptosis levels, and the functional impairment. Notably, we proved that the inhibition of PKM2 demonstrated potential in mitigating these injuries and elucidated possible mechanisms.

## 2 Materials and Methods

### 2.1 Animals

The Sprague Dawley rats used in this study were sourced from the East Campus of the Laboratory Animal Center at Sun Yat‐sen University, with the license number SCXK (GD) 2021‐0029. The trials were carried out using neonatal rats at the age of 7 days and random assignment of gender. The animal study methodologies used in this research were granted approval by the Institutional Animal Care and Use Committee at the National Institutes of Health, Sun Yat‐sen University, by the ethics approval No. SYSU‐IACUC‐2023‐001814. The experimental model used to induce HIBD in newborn rats was established via the utilization of the modified Rice–Vannucci technique (Rice, Vannucci, and Brierley 1981). We performed Rice–Vannucci ligation of the left common carotid artery in the anesthetized HIBD group of rats and placed them in an 8% O_2_ equilibrated with N_2_ hypoxic chamber for 2.5 h after 1.5 h of postoperative recovery. In the sham‐operated group, only a neck skin incision was made, as well as isolation of the left common carotid artery, and the rats were shifted to a normoxic environment after the operation without hypoxia. The rats were randomly divided into four groups using a random allocation method: the sham group, which was operated on but without ligation; the Shikonin group, which was operated on but without ligation and injected with a 25 mg/kg dose of Shikonin (C.I. 75535 from Selleck, China) dissolved in 1% DMSO intraperitoneally once daily for 3 days; the HIBD group; the Shikonin + HIBD group, which consisted of HIBD modeling rats injected with shikonin. After 72 h of HIBD modeling, behavioral tests were conducted, followed by anesthesia and euthanasia of all rats to collect brain and blood samples.

### 2.2 Neurological Deficit Assessment and Behavioral Analysis

A neurobehavioral assessment was performed 72 h post‐surgery utilizing the geotaxis test and righting reflex test. In the righting reflex test, neonatal rats were positioned in a dorsal downward orientation with their limbs facing upward, supine on an operating table, and the time required to turn upright was recorded. The geotaxis test, in which rats were placed head down on a slope and the time taken to turn the body in an upward direction, was measured.

### 2.3 Hematoxylin–Eosin Staining

Brain tissues were collected from rats that were deeply anesthetized and euthanized. The collected tissues were then fixed using a 4% paraformaldehyde (PFA) solution, dehydrated, embedded in paraffin, and sectioned. Subsequently, the sections were deparaffinized in xylene for approximately 5 min and rehydrated by using different concentrations of alcohol. Following this, hematoxylin staining was performed for approximately 6 min, followed by a 30‐s differentiation step in the presence of 1% hydrochloric acid alcohol. The sections were then briefly exposed to 0.5% ammonia to restore the blue color and subsequently stained with eosin for approximately 20 s. The specimen underwent a staining process involving hematoxylin for approximately 6 min, followed by differentiation using 1% hydrochloric acid alcohol for approximately 30 s. Subsequently, eosin staining was performed for approximately 30 s, and 0.5% ammonia was applied to restore the blue color after 30 s. After 1 min, each step was subjected to a gradient of ethanol and xylene, and the resulting images were sealed using neutral resin glue. Subsequent microscopic examination of the hippocampus revealed the presence of pathological alterations.

### 2.4 Immunofluorescence

The rat brains were fixed in a 4% PFA solution at a temperature of 4°C. Subsequently, the brains underwent a process of dehydration, were then fixed in paraffin, and then sectioned. The sections were then subjected to overnight incubation at a temperature of 4°C with primary antibodies targeting PKM2 (YT3777, 1:200; Immunoway, China), NeuN (26975‐1‐AP, 1:500; Proteintech, China), Iba‐I (81728‐1‐RR, 1:100; Proteintech, USA), and Cleaved Caspase‐1 (1:200; Immunoway, China). After incubation with Alexa Fluor‐FITC/CY3/CY5 secondary antibodies protected from light for 1 h, DAPI was used to restain the sections. The immunofluorescence pictures were obtained using an Olympus BX63 fluorescent microscope, and the positive signals were evaluated using ImageJ software.

### 2.5 Lactate Dehydrogenase Release Assay

Lactate dehydrogenase assay kit (A020‐1‐2; Nanjing Jiancheng, China). The quantification of lactate dehydrogenase (LDH) release was conducted by determining the proportion of LDH present in the serum, expressed as a percentage of the total LDH content, using a wavelength of 450 nm.

### 2.6 ELISA of Inflammatory Cytokines

Samples of arterial blood were obtained from every group. The samples underwent centrifugation at a speed of 3000 revolutions per minute for 20 min, followed by storage at a temperature of −80°C for future use. To quantify the concentrations of IL‐18 and IL‐1β, we used particular enzyme‐linked immunosorbent assay (ELISA) kits (Elabscience, Wuhan, China).

### 2.7 Western Blot

PKM2 and pyroptosis‐related proteins were examined via Western blotting using lysates from rat brains, and an Assay kit BCA was used to measure protein concentrations (Beyotime, Shanghai, China). To analyze the lysed proteins further, SDS‐PAGE gels were separated, and the gels were transferred onto nitrocellulose membranes (Millipore, USA). The membrane was blocked with a 5% bovine serum albumin solution and subjected to incubation at a temperature of 4°C, in the presence of the subsequent primary antibodies.: anti‐Cleaved Caspase‐1 p20 (D210) (YC0002, 1:1000), NLRP 3 (YT5382, 1:1000; Immunoway), PKM2 (YT3777, 1:1000; Immunoway), and GSDMD n‐ternal (YT7991, 1:1000; Immunoway), IL‐1β (YT5201, 1:1000; Immunoway), β‐actin (K010174M‐Biotin, 1:1000; solarbio). The primary antibodies were subjected to overnight incubation in the presence of antibodies. Following three rounds of washing in TBST, the samples were subjected to a 1‐h incubation at ambient temperature with the secondary antibody linked to horseradish peroxidase. The analysis of the utilization of the enhanced chemiluminescence detection method (Syngen G: BOX Chemi XT4) was performed using ImageJ software.

### 2.8 Statistics

The statistical analyses were performed utilizing GraphPad Prism 8.0 software, which computed the means and standard errors of the mean (SEM). The statistical analyses employed in this investigation encompassed the utilization of one‐way analysis of variance (one‐way ANOVA) and Sidak's multiple comparisons test to conduct comparisons. A significant threshold of *p *< 0.05 was employed to ascertain statistical significance.

### 2.9 PKM2 Expression Is Increased in Hippocampal Tissues of HIBD Rats

**FIGURE 1:**
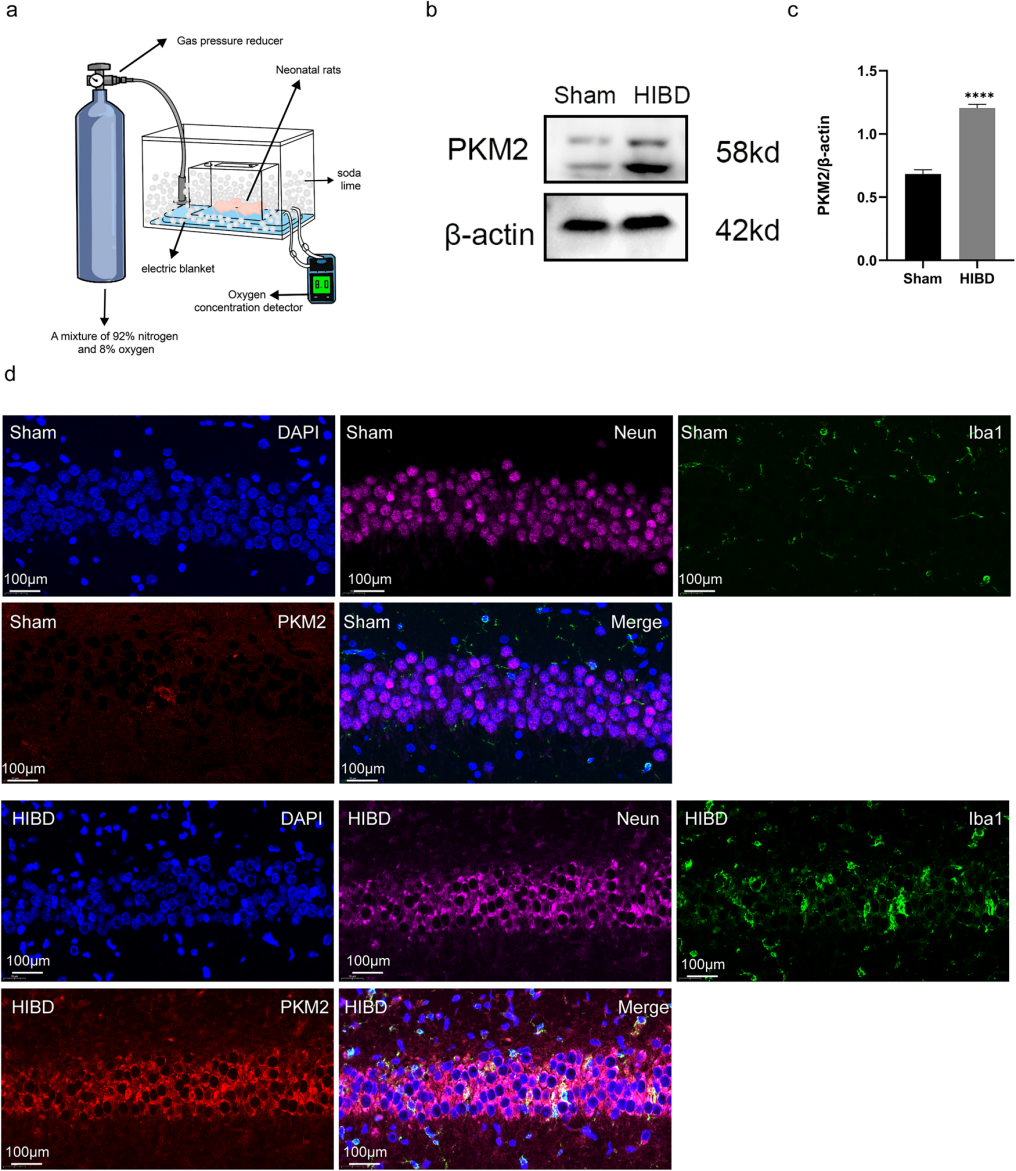
PKM2 expression is increased in hippocampal tissues of HIBD rats. (a) Western blot was utilized to assess the expression of PKM2 (*n* = 6). (b) Gray scale statistics were obtained to determine the levels of PKM2 (*n* = 6). (c) The localization of PKM2 in hippocampal tissue slices of rats in the HIBD group was observed through triple staining of PKM2/Iba1/Neun fluorescence. *p < 0.05, **p < 0.01, ***p < 0.001, ****p < 0.0001.

In order to investigate the protein expression level of PKM2 in the rat brain following the HIBD model, a western blot assay was conducted on hippocampal tissues from both sham and surgical rats. The results indicated a significant increase in PKM2 levels in the hippocampus of the HIBD group 72 h after surgery (*p* < 0.0001). In addition, to determine the localization of PKM2 in brain tissue cells, a PKM2/Neun/Iba1 fluorescence triple labeling was performed on hippocampal tissue sections from both the sham and HIBD groups. The findings revealed an elevation in PKM2 expression in the hippocampal tissues of the HIBD group. And this elevation was mainly observed in neuronal cells rather than microglia (Figure 1).

### 2.10 Inhibition of PKM2 Demonstrated Efficacy in Ameliorating Behavioral Deficits Following Cerebral Ischemia‐Hypoxia Injury in Neonatal Rats

**FIGURE 2:**
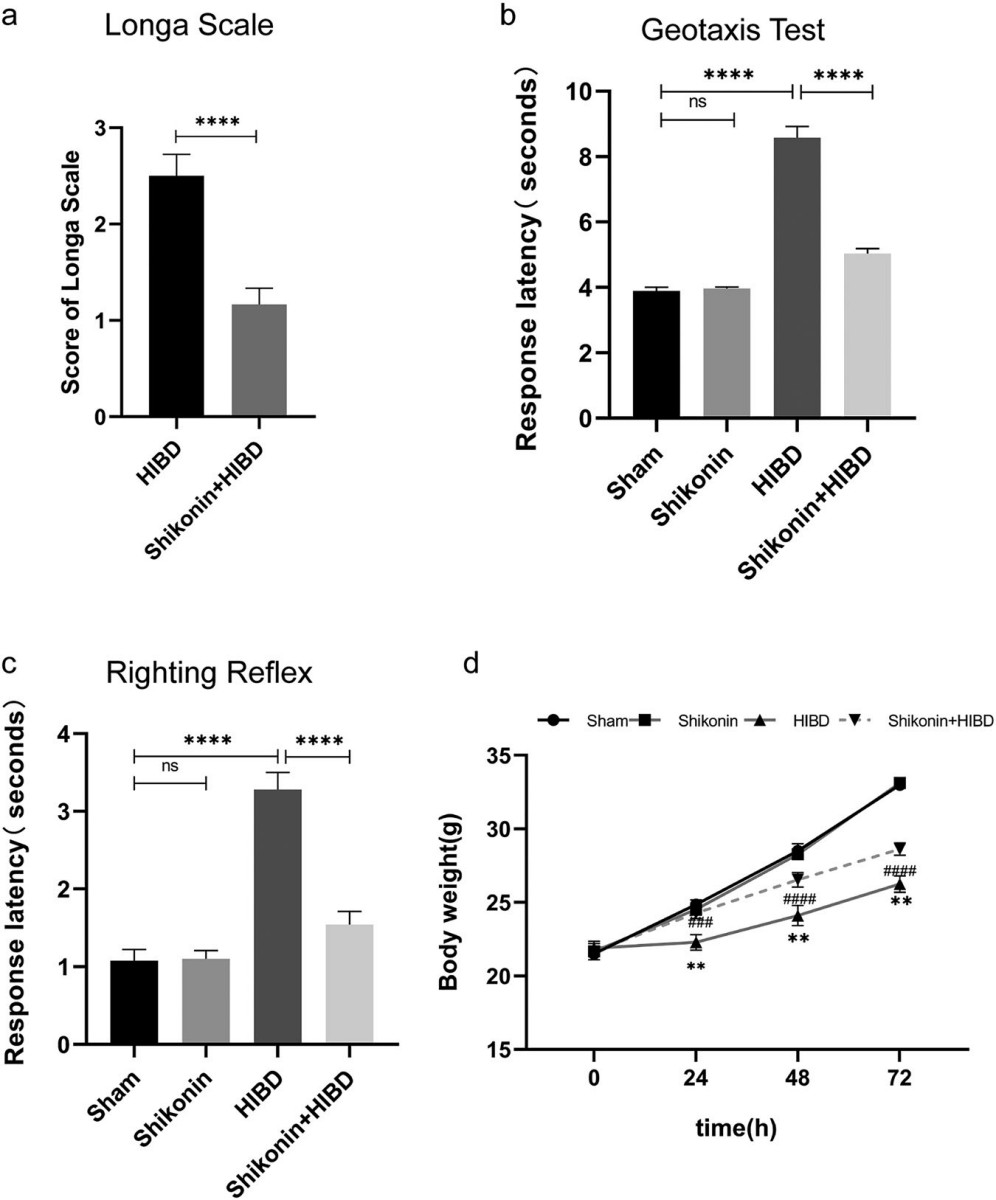
Inhibition of PKM2 has been found to enhance weight gain and ameliorate neurobehavioral deficits in rats with HIBD. (a) The geotaxis test (*n* = 6) was performed on the four groups. (b) The righting reflex (*n* = 6) was evaluated in the rats. (c) Body weight measurements were recorded within 72 h for the four groups of rats (*n* = 6). *p < 0.05, **p < 0.01, ***p < 0.001, ****p < 0.0001 vs. sham; ^#^ *p* < 0.05, ^##^ *p* < 0.01, ^###^ *p* < 0.001, ^####^ *p* < 0.0001 vs. HIBD.

The Rice–Vannucci model was utilized for HIBD modeling, resulting in a range of symptoms in the HIBD group, including irritability, cyanosis, and lethargy. Conversely, the Shikonin + HIBD group exhibited significant improvement in irritability and cyanosis compared to the HIBD group post‐modeling. The body weights of the four rat groups were assessed at multiple time points during the initial 72‐h period after the hypoxic‐ischemic insult. The results showed that the HIBD group experienced significantly less weight gain three days after surgery than the sham group (all *p* < 0.01) (Figure 2c). The difference in body weight between the sham and shikonin alone groups was not significant. To evaluate the neurological function of the four groups of rats, we used the geotaxis test and righting reflex, it was observed that the duration of both tests was longer in the HIBD group than in the sham group (*p* < 0.0001). Consequently, the difference in duration between the sham group and the shikonin alone group was not significant. Nevertheless, the administration of shikonin led to a substantial decrease in this prolonged duration (*p* < 0.0001; Figure 2a,b). These findings suggest that the inhibition of PKM2 effectively alleviates motor nerve injury in neonatal rats following ischemia and hypoxia, leading to improvements in weight gain and neurobehavioral deficits in HIBD rats.

### 2.11 Inhibition of PKM2 Exhibited a Mitigating Effect on the Pathology of Cerebral Ischemic‐Hypoxic Injury

**FIGURE 3:**
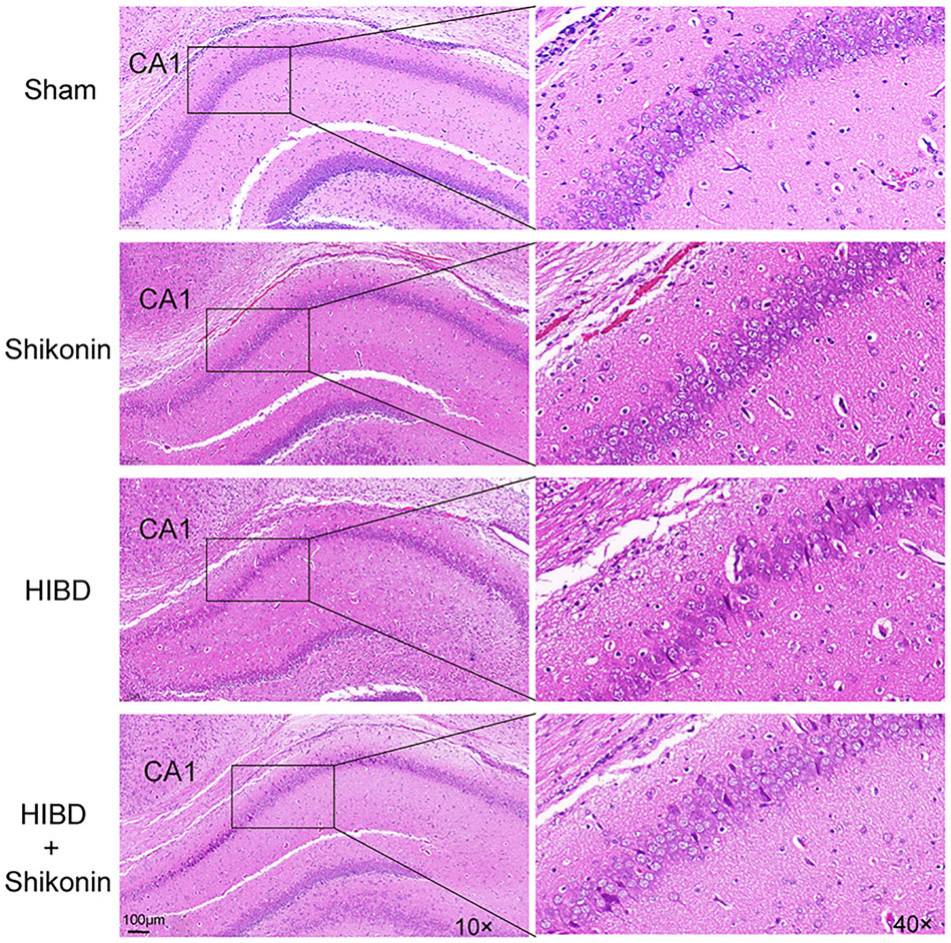
Inhibition of PKM2 effectively mitigated pathological damage in neonatal rats subjected to cerebral ischemia‐hypoxia injury HE staining, which allowed for the assessment of pathological damage in hippocampal CA1 neurons in each group of HIBD rats.

The extent of cerebral injury was evaluated in four cohorts of rats following 72 h of ischemia‐hypoxia. Figure 3 depicts the histological examination of hippocampal sections, wherein the nuclei of cells are stained blue and the cytoplasm appears pink or red. There was no significant difference in hematoxylin–eosin staining between the sham group and the shikonin group alone. Hippocampus nerve cells of the HIBD group had conspicuous damage, characterized by reduced numbers, disorganized arrangement, and indistinct boundaries. In addition, peripheral regions displayed substantial infiltration of inflammatory cells and proliferation of glial cells. Some cells exhibited uneven staining and nuclear hyperchromatosis, characteristic of hypoxic‐ischemic pathological injury. Following intraperitoneal administration of shikonin, there was a notable alleviation of the pathological injury, a significant reduction in neuronal cell loss and damage, and a more regular arrangement of cells. These findings provide evidence that the inhibition of PKM2 effectively safeguards neurons in the hippocampus regions of rats with hypoxic‐ischemic injuries.

### 2.12 Inhibition of PKM2 Demonstrated a Mitigating Effect on the Acute Inflammatory Response

**FIGURE 4:**
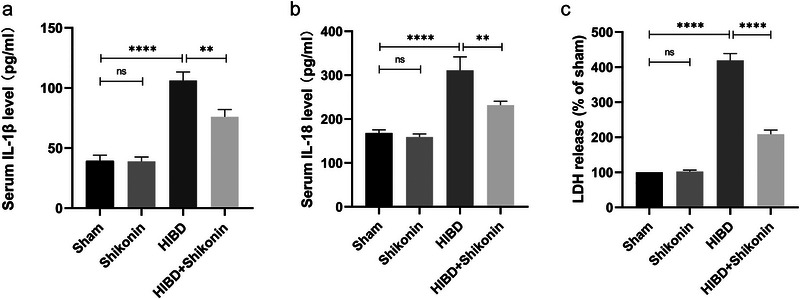
Inhibition of PKM2 attenuated acute inflammatory response in cases of HIBD. (a, b) ELISA using a rat serum IL‐1β and IL‐18 inflammatory factors kit to assess the levels of these factors (*n* = 6). (c) Measuring lactate dehydrogenase levels in serum (*n* = 6). *p < 0.05, **p < 0.01, ***p < 0.001, ****p < 0.0001.

To examine the impact of PKM2 inhibiting on neonatal rats with ischemic‐hypoxic brain injury in terms of acute inflammation, ELISA kits were initially utilized to detect the presence of IL‐18 and IL‐1β inflammatory components in the blood of four rat groups. The results of the study demonstrated significantly elevated levels of IL‐1β and IL‐18 inflammatory components in the HIBD group compared to the sham‐operated group (Figure 4a,b). In addition, the injection of shikonin‐reduced LDH levels significantly (*p* < 0.0001) (Figure 4c).

### 2.13 Inhibition of PKM2 Demonstrated Inhibitory Alleviate Neuronal Pyroptosis in Hippocampus by Suppressing the NLRP3/Caspase‐1/GSDMD Pathway

**FIGURE 5:**
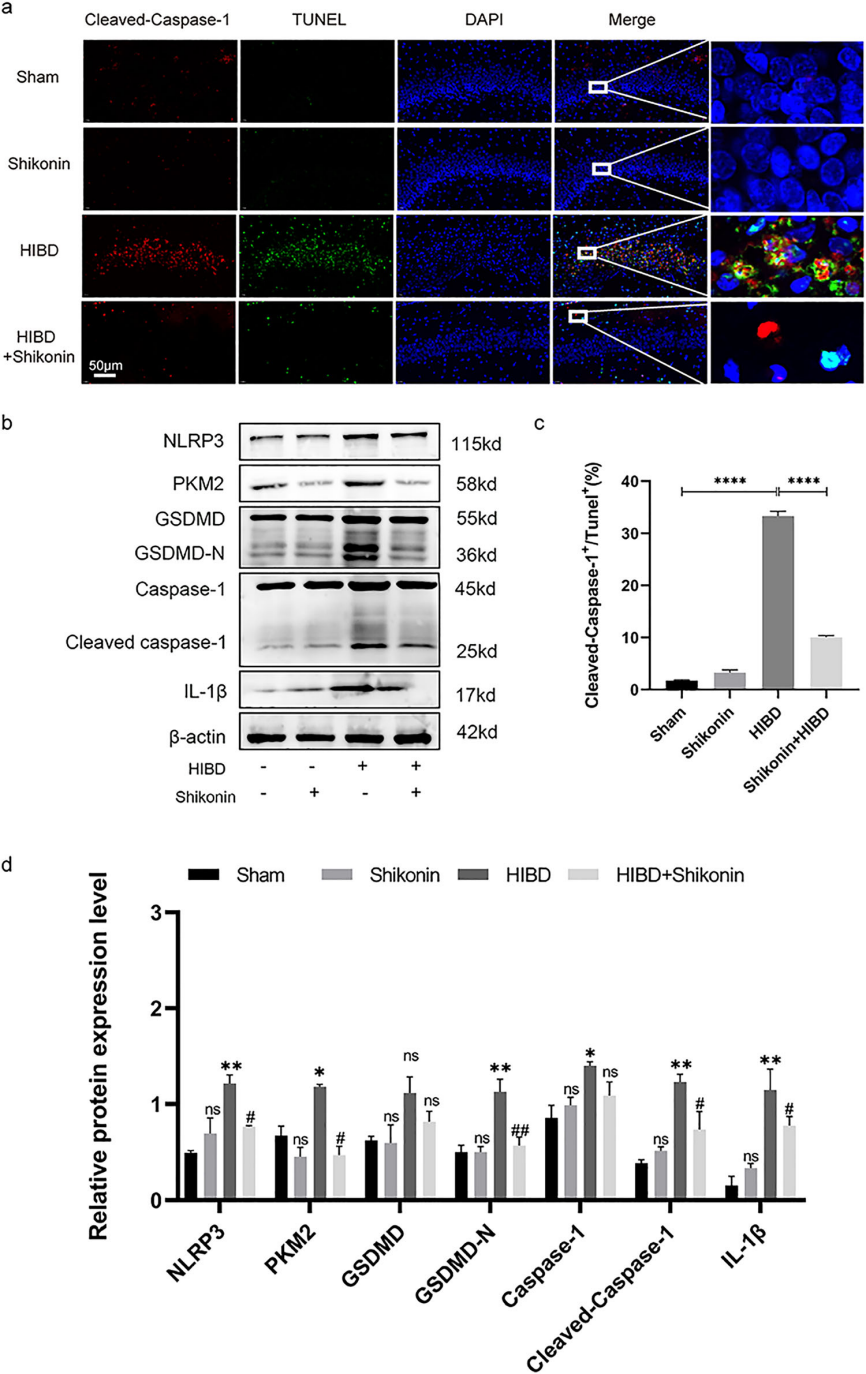
Inhibition of PKM2 demonstrated inhibitory effects on PKM2 and NLRP3 expression, alleviate suppressing neuronal pyroptosis. (a) Double staining with TUNEL^+^ and Cleaved Caspase‐1^+^ to assess the percentage of damaged neuronal cells in the hippocampus regions of HIBD rats in each group (*n* = 6). (b) Western blot was utilized to assess the expression of PKM2, NLRP3, Caspase‐1, Cleaved Caspase‐1, GSDMD, GSDMDN, and IL‐1β in the hippocampus of rats that underwent HIBD in four separate groups (*n* = 3). (c) The statistical representation of pyroptosis cells (%) = (TUNEL^+^/Cleaved Caspase‐1^+^ cells)/total cells × 100% (*n* = 6). (d) Gray scale statistics were obtained to determine the levels of PKM2, NLRP3, Caspase‐1, Cleaved Caspase‐1, GSDMD, GSDMDN, and IL‐1β expression in the hippocampus of rats across the four experimental groups (*n* = 3). *p < 0.05, **p < 0.01, ***p < 0.001, ****p < 0.0001 vs. sham group; ^#^ *p* < 0.05, ^##^ *p* < 0.01, ^###^ *p* < 0.001, ^####^ *p* < 0.0001 vs. HIBD.

In this study, brain sections from four groups of neonatal suckling rats were subjected to TUNEL/Cleaved Caspase‐1 fluorescence staining. Pyroptotic cells were identified as TUNEL^+^/Cleaved Caspase‐1^+^ cells (Figure 5a). The observed fraction of pyroptotic cells in the HIBD group demonstrated a statistically significant elevation in comparison to the sham‐operated group. Nonetheless, the injection of shikonin led to a significant decrease in the percentage of pyroptotic cells (*p* < 0.0001) (Figure 5d). In addition, Western blot tests conducted on the hippocampus revealed a significant decrease in the expression of PKM2, NLRP3, Caspase‐1, Cleaved Caspase‐1, GSDMD, GSDMDN, and IL‐1β in the group treated with shikonin in comparison to the group subjected to HIBD (all *p* < 0.05) (Figure 5b,d). These findings suggest that inhibiting PKM2 can ameliorate hippocampal neuronal pyroptosis induced by HIBD.

## 3 Discussion

Pyruvate kinase isoform M2 (PKM2) is a significant indicator of neuronal cellular responses to OGD/R (Shirai et al. 2016). In addition, PKM2 triggers the assembly of the inflammasome component PKR (also known as EIF2AK2) in a phosphorylated state, which is reliant on lactate. The phosphorylated state of PKR is of significant importance in promoting the production of inflammatory vesicles by NLRP3 and ASC (Lin and Mei 2020). Furthermore, the involvement of the NLRP3 inflammasome has been implicated in the phenomenon of neuroinflammation. Upon initiation, the NLRP3 inflammasome induces the enzymatic separation of pro‐IL‐1β and pro‐IL‐18 into their fully developed states through the involvement of Caspase‐1. Caspase‐1 can enzymatically cleave gasdermin D (GSDMD), triggering the process of cellular pyroptosis (Zhang et al. 2020).

In our investigation, we observed a predominant upregulation of PKM2 in neurons rather than microglia in the hippocampal regions of the brain affected by injury. In addition, we identified an increase in the expression of inflammatory factors IL‐1β and IL‐18, as well as elevated serum LDH levels following HIBD in rats. Conversely, the inhibition of PKM2 effectively suppressed the expression of post‐HIBD proteins GSDMD‐N, Cleaved Caspase‐1, and NLRP3. At the same time, we tested the neonatal rats by the righting reflex experiment (72 h after modeling); the neonatal rats were gently flipped over on the manipulator so that their backs were facing downward and their limbs were facing upward. Subsequently, the rats were supported with one hand and held in a supine position for about 2 s before releasing the hand, and timing was started immediately. Observe and record the time required for the rat to return from the supine position to the normal standing position and geotaxis test (72 h after modeling); neonatal rats were inverted head to tail and placed on a 45° rough slope surface. Then, the time required for the rats to complete the 180° turning maneuver was observed and recorded (Altman and Sudarshan 1975; Malinová‐Ševčíková et al. 2014; Hrubá et al. 2009). Behavioral assessment of rats in each group showed that inhibition of PKM2 effectively alleviated motor nerve injury after ischemia and hypoxia in neonatal rats, thereby improving growth and development and neurobehavioral deficits in HIBD rats.

Based on these findings, we propose the hypothesis that PKM2 plays a crucial role in the regulation of neuronal pyroptosis after HIBD in rats. This study establishes a connection between the metabolism‐related protein PKM2 and the cellular pyroptosis network. It is evident that metabolic alterations and the initiation of cellular pyroptosis are interdependent, suggesting a potential correlation between them. This noteworthy phenomenon suggests the existence of a reciprocal regulation mechanism between metabolic regulation and cellular pyroptosis. The findings of this study offer valuable preliminary insights for future investigations into the explicit regulatory mechanisms governing the relationship between these two processes.

However, it is important to acknowledge that PKM2 exists in two distinct active states, namely dimeric and tetrameric. Notably, the cytoplasmic localization of the tetrameric configuration of PKM2 is mostly observed in rapidly proliferating cells (Liu et al. 2021; Zhang et al. 2019). Primarily acting as a protein kinase to stimulate HIF‐1α, the dimeric form helps proinflammatory macrophages and cancer cells undergo aerobic glycolytic metabolic reprogramming. (Shirai et al. 2016; Palsson‐McDermott et al. 2015). Huang et al. conducted a study demonstrating that shikonin displays nonspecific inhibitory effects on both active forms (Huang et al. 2022). However, the exact influence of this inhibition on the aggregation state cannot be conclusively determined, thus requiring further investigation into the mechanism through which PKM2 operates in the context of HIBD neurological damage.

## 4 Conclusion

The findings of this study indicate that the suppression of PKM2 holds promise for enhancing the neurological function of neonatal rats afflicted with ischemic‐hypoxic brain injury. Additional investigation is warranted to explore the underlying mechanisms linking PKM2 with NLRP3/Caspase‐1/GSDMD, which play a role in mediating the safeguarding effects against neuronal damage and pyroptosis arising from HIBD.

## Author Contributions

**Sha Sha**: conceptualization, methodology, investigation, formal analysis, writing–original draft. **Ni Jin**: investigation, formal analysis, writing–review and editing. **Ruiyu Zhou**: writing–review and editing. **Yanghao Ruan**: writing–review and editing. **Ying Ouyang**: conceptualization, funding acquisition, resources, supervision. All authors contributed to the article and approved the submitted version.

## Conflicts of Interest

The authors declare no conflicts of interest.

## Ethics Statement

The animal study methodologies used in this research were granted approval by the Institutional Animal Care and Use Committee at the National Institutes of Health, Sun Yat‐sen University, by the Ethics Approval No. SYSU‐IACUC‐2023‐001814

### Peer Review

The peer review history for this article is available at https://publons.com/publon/10.1002/brb3.70108.

## Data Availability

The data that support the findings of this study are available from the corresponding author upon reasonable request.
